# Emerging Technologies for Real‐Time Intraoperative Margin Assessment in Future Breast‐Conserving Surgery

**DOI:** 10.1002/advs.201901519

**Published:** 2020-03-17

**Authors:** Ambara R. Pradipta, Tomonori Tanei, Koji Morimoto, Kenzo Shimazu, Shinzaburo Noguchi, Katsunori Tanaka

**Affiliations:** ^1^ Biofunctional Synthetic Chemistry Laboratory RIKEN Cluster for Pioneering Research 2‐1 Hirosawa Wako Saitama 351‐0198 Japan; ^2^ School of Materials and Chemical Technology Department of Chemical Science and Engineering Tokyo Institute of Technology 2‐12‐1 Ookayama, Meguro‐ku Tokyo 152‐8552 Japan; ^3^ Department of Breast and Endocrine Surgery Graduate School of Medicine Osaka University 2‐2‐E10 Yamadaoka, Suita Osaka 565‐0871 Japan; ^4^ Biofunctional Chemistry Laboratory A. Butlerov Institute of Chemistry Kazan Federal University 18 Kremlyovskaya Street Kazan 420008 Russia; ^5^ GlycoTargeting Research Laboratory RIKEN Baton Zone Program 2‐1 Hirosawa Wako Saitama 351‐0198 Japan

**Keywords:** artificial intelligence algorithms, breast cancer, breast‐conserving surgery, deep learning, imaging, intraoperative diagnosis

## Abstract

Clean surgical margins in breast‐conserving surgery (BCS) are essential for preventing recurrence. Intraoperative pathologic diagnostic methods, such as frozen section analysis and imprint cytology, have been recognized as crucial tools in BCS. However, the complexity and time‐consuming nature of these pathologic procedures still inhibit their broader applicability worldwide. To address this situation, two issues should be considered: 1) the development of nonpathologic intraoperative diagnosis methods that have better sensitivity, specificity, speed, and cost; and 2) the promotion of new imaging algorithms to standardize data for analyzing positive margins, as represented by artificial intelligence (AI), without the need for judgment by well‐trained pathologists. Researchers have attempted to develop new methods or techniques; several have recently emerged for real‐time intraoperative management of breast margins in live tissues. These methods include conventional imaging, spectroscopy, tomography, magnetic resonance imaging, microscopy, fluorescent probes, and multimodal imaging techniques. This work summarizes the traditional pathologic and newly developed techniques and discusses the advantages and disadvantages of each method. Taking into consideration the recent advances in analyzing pathologic data from breast cancer tissue with AI, the combined use of new technologies with AI algorithms is proposed, and future directions for real‐time intraoperative margin assessment in BCS are discussed.

## Graphical abstract

Clean surgical margins in breast‐conserving surgery (BCS) are essential for preventing recurrence and repeat surgery. This work describes the advantages and disadvantages of conventional pathologic and newly developed techniques. Future directions and prospectives in real‐time intraoperative margin assessment in BCS as well as recent advances in deep learning and artificial intelligence algorithms are discussed.

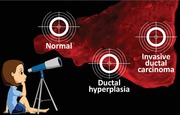

## 1 Introduction

Breast cancer is a highly prevalent disease that affects many women worldwide.^[^ ^1^ ^]^ For women with early‐stage breast cancer, breast‐conserving surgery (BCS) is the standard therapy. Moreover, a meta‐analysis by the Early Breast Cancer Trialists' Collaborative Group showed that postoperative radiation therapy reduces the risk of ipsilateral breast tumor recurrence (IBTR).^[^ ^2^ ^]^ However, as suggested by the Society of Surgical Oncology and the American Society for Radiation Oncology, surgical margins are also significant. In their consensus guidelines, they stressed the importance of managing margins for BCS in Stages I and II patients.^[^ ^3^ ^]^ A surgical margin is defined as negative if no malignant cells are observed at the surface of the resected invasive cancer specimen. “No tumor on ink” is used to indicate that the inked borders are free from any detectable tumor tissue. In addition, the consensus guidelines recommend a clear margin of 2 mm in the case of ductal carcinoma in situ (DCIS),^[^ ^3^ ^]^ pointing out the importance of diagnosing cancerous breast tissue morphology for both invasive cancer and DCIS.

Recently, a meta‐analysis of 28 162 patients from 33 studies reported that positive margins analyzed using postoperative pathologic methods are associated with at least a twofold increase in IBTR. On the other hand, negative margins are associated with low rates of IBTR and have the potential to decrease re‐excision rates.^[^ ^4^ ^]^ However, there is currently no established global standard for real‐time and fast intraoperative margin management in BCS.

**Figure 1:**
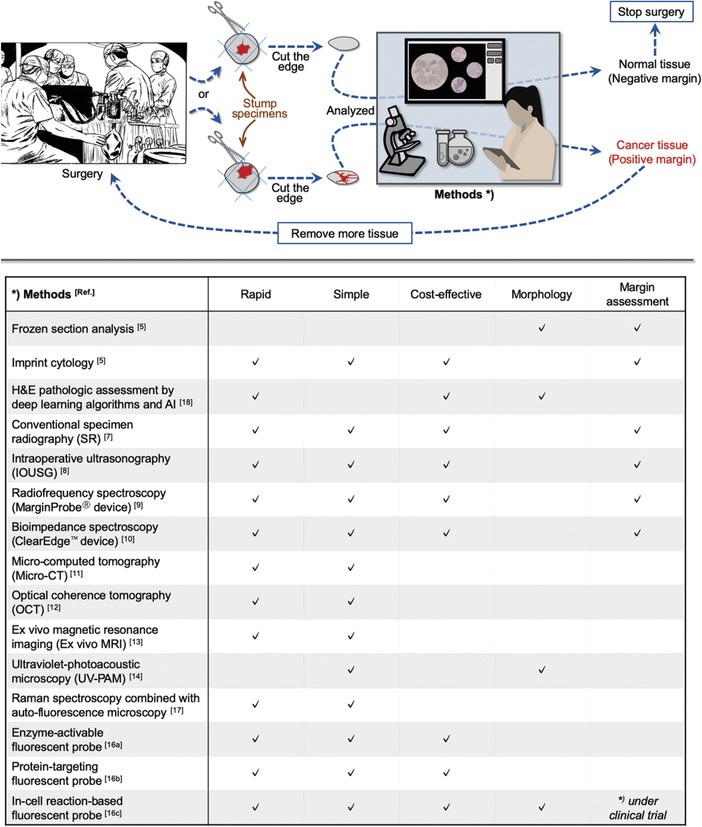
Schematic of intraoperative diagnosis methods. Frozen section analysis has been used for decades as the gold standard during breast‐conserving surgery (BCS). Despite its reliability, this traditional method is complicated and time‐consuming. Researchers have made substantial efforts to develop techniques for quicker and simpler diagnosis of cancerous breast tissue. However, most of the technologies that have recently emerged still have challenges in providing fast, simple, and cost‐effective diagnosis, which includes visualization of the morphology of cancerous breast tissue. Meanwhile, very recently, we reported an in‐cell reaction‐based fluorescent probe for diagnosing cancerous breast tissue morphology in a rapid, simple, and cost‐effective procedure. The efficiency of newly emerging methods in the category column (rapid, simple, cost‐effective, morphology, margin assessment) was evaluated by comparison with frozen section analysis.

Intraoperative pathologic methods, which include frozen section analysis and imprint cytology, are the traditional choices for intraoperative diagnosis during BCS.^[^ ^5^ ^]^ These methods have the potential to lower rates of positive margins (**Figure** **1**).^[^ ^6^ ^]^ However, in order to generalize the method, we need to solve several problems, such as the complexity and time‐consuming nature of these pathologic procedures and the demanding workload placed on pathologists. Recently, researchers have been seeking new techniques. Several methods have recently emerged for real‐time intraoperative management of breast margins using live tissue. These methods include conventional specimen radiography (SR),^[^ ^7^ ^]^ intraoperative ultrasonography (IOUSG),^[^ ^8^ ^]^ radio‐frequency spectroscopy (MarginProbe device),^[^ ^9^ ^]^ bioimpedance spectroscopy (ClearEdge device),^[^ ^10^ ^]^ microcomputed tomography (micro‐CT),^[^ ^11^ ^]^ optical coherence tomography (OCT),^[^ ^12^ ^]^ ex vivo magnetic resonance imaging (ex vivo MRI),^[^ ^13^ ^]^ ultraviolet photoacoustic microscopy (UV‐PAM),^[^ ^14^ ^]^ microscopy with ultraviolet surface excitation (MUSE),^[^ ^15^ ^]^ chemistry‐based fluorescent probes,^[^ ^16^ ^]^ and a multimodal imaging technique combining tissue autofluorescence and Raman spectroscopy with both macro (tissue‐level) and micro (cell‐level) detection.^[^ ^17^ ^]^

In this Review, we describe the traditional pathologic and newly developed techniques. We discuss the advantage and disadvantages of each method. We highlight clinical needs and potential value in terms of margin management in breast cancer. Taking into consideration the recent advances in analyzing pathologic data in breast cancer tissue with deep learning models,^[^ ^18^ ^]^ we propose the combined use of new technologies with artificial intelligence (AI) algorithms. We also discuss future directions and prospects for real‐time intraoperative margin assessment in BCS.

## 2 Pathologic Methods

Frozen section analysis and imprint cytology are the traditional pathologic methods for real‐time intraoperative margin assessment in BCS.^[^ ^5^ ^]^ Many intraoperative examples have been reported for pathologic analysis. These methods have the highest diagnostic accuracy in terms of both sensitivity and selectivity and are currently recognized as the most promising methods for lowering the rates of positive margins during BCS.^[^ ^6^ ^]^ The reoperation rate for patients who undergo imprint cytology or intraoperative frozen section margin assessment is lower than that of patients who do not receive any intraoperative margin status assessment.^[^ ^5^, ^19^ ^]^ In addition, the routine use of intraoperative frozen section analysis can be cost‐effective for both the patient and the hospital.^[^ ^20^ ^]^

### 2.1 Frozen Section Analysis

In rapid frozen section analysis, breast tissue samples, i.e., stumps specimens, from the surgical resection are embedded in the optimal cutting temperature compound, frozen, and cut into slices. The slices are then put on a glass slide and fixed with paraformaldehyde for immunohistochemical staining. Next, light microscopy of sections stained with hematoxylin and eosin (H&E) are used to perform the pathologic evaluation. A significant advantage of H&E staining analysis of the frozen section is that this method can determine the presence of cancer in the surgical samples of interest as well as diagnose the type of various cancers. Thus, further surgery could be guided by whether morphological analysis shows that the sample consists of DCIS, invasive ductal carcinoma (IDC), ductal hyperplasia (DH), or normal breast gland (NBG).

Nevertheless, currently these methods are not used for BCS in hospitals worldwide. Reasons include complicated sample preparation, which requires an additional skilled technician to cut the specimens for frozen section interpretation and tedious pathologic analysis that requires ≈30 min for a single assessment.^[^ ^5b,c,e^ ^]^ If additional examination is needed, tremendous efforts and amounts of time are necessary. A worldwide shortage of trained pathologists, which is more severe than the shortage of physicians in general, is another limiting factor for routine frozen section analysis as part of intraoperative margin assessment in BCS.

### 2.2 Imprint Cytology

Imprint cytology analysis, on the other hand, is a simpler method used during BCS in some hospitals. Live tissue samples are rubbed onto a glass slide. The attached cells are immediately fixed with ethanol. Next, H&E or Papanicolaou staining is performed on the ethanol‐fixed cells. Imprint cytology analysis is based on the idea that malignant cells will adhere to the slides, whereas adipose cells will not. Imprint cytology analysis examines the entire surface of the resected tissue, unlike the spot checks that occur with frozen section analysis.

Many reports on BCS show that imprint cytology can achieve similarly high diagnostic accuracy as frozen section analysis.^[^ ^5a,d,e^ ^]^ However, this method can only determine the presence of cancer. It cannot analyze morphology. Rapid and accurate interpretation requires a professional trained in cytopathology in the operating room in addition to the regular surgical team.^[^ ^21^ ^]^

The increase in operative time resulting from the pathologic procedure and the increased workload for pathologists inhibit the broader applicability of this method as an established global standard for intraoperative diagnosis. Thus, although frozen section analysis and imprint cytology are the most reliable methods currently available, only a limited number of hospitals in some developed countries actually use them for rapid intraoperative diagnosis during BCS.

### 2.3 H&E Pathologic Assessment by Deep Learning and AI Algorithms

To circumvent the shortage of and workload burden for pathologists and improve the diagnostic accuracy of intraoperative margin assessment in BCS, significant attention has been focused on automated deep learning algorithms. An exciting international competition (CAMELYON16) to assess the effectiveness of automated deep learning algorithms in diagnosing the H&E sections of axillary lymph node metastasis was conducted during November 2015 to November 2016.^[^ ^18a^ ^]^ Thirty‐two algorithms and 12 pathologists (with and without time constraints) were tested with 129 whole‐slide images (49 with and 80 without metastasis); the task was to classify images as definitely normal tissue, probably normal tissue, equivocal, probably tumor, or definitely tumor.

The algorithms developed by Harvard Medical School and Massachusette Institute of Technology achieved the highest score (true‐positive fraction, 72.4%), which was comparable to scores from pathologists without time constraints. It is noteworthy to mention that the best algorithms, i.e., those with an area under the receiver operating characteristic curve (AUC) of 0.994, performed significantly better than pathologists when there is time constraint for diagnosis (AUC, 0.810). The top five algorithms had a mean AUC of 0.960, which was comparable with pathologists without time constraints (AUC, 0.966).

These data showed that the deep learning algorithms exhibited better diagnostic performance than 12 pathologists taking part in a simulation exercise designed to mimic the routine pathology workflow for axillary lymph nodes. Alternatively, the performance of the algorithms was comparable with an expert pathologist interpreting images with H&E staining under no time constraints. Thus, deep learning algorithms have significant potential to circumvent the problems associated with pathologic methods. However, other than for axillary lymph nodes in breast cancer, there have been no reports to date on using new algorithms to interpret pathologic information for margin assessment in BCS. Alternatively, we could combine these AI algorithms with other newly emerging rapid and convenient techniques that could give the same pathologic information for diagnosis during BCS in the future.

Recently, Tsirigos and co‐workers developed a deep learning model using publicly available whole‐slide images in the Cancer Genome Atlas to accurately and automatically classify histopathologic images of non‐small cell lung cancer from different cohorts collected at their institution.^[^ ^18b^ ^]^ They demonstrated that a convolutional neural network, such as Google's inception v3, can be used to assist in the diagnosis of lung cancer from histopathologic slides, reaching sensitivity and specificity comparable to that of a pathologist. Furthermore, by analyzing only the pathology images, the network was also able to predict the most commonly mutated genes in lung adenocarcinoma. These findings suggest that deep learning and AI algorithms have the potential to predict gene mutations in various kinds of cancers.

## 3 Conventional Imaging Methods

Although BCS has been used as the primary treatment for early‐stage breast cancer, more accurate techniques are needed to assess resection margins during surgery to avoid the need for re‐excision and reoperation. Intraoperative specimen imaging methods such as conventional SR and IOUSG provide timely information on whether re‐excision of a cavity shave margin is indicated during routine BCS.

### 3.1 Conventional Specimen Radiography (SR)

**Figure 2:**
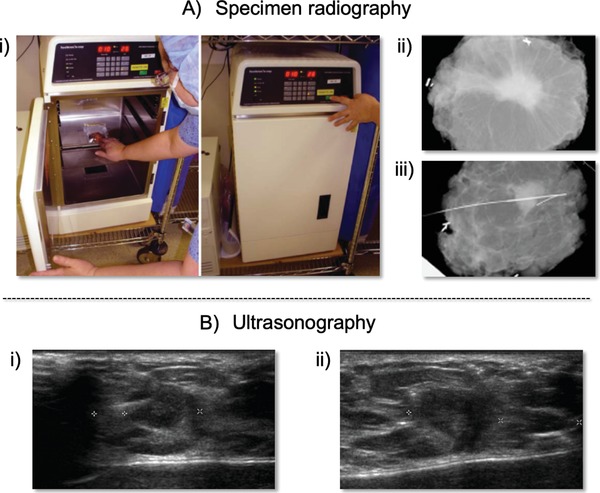
A‐i) Digital specimen mammography device for identifying lumpectomy targets during breast‐conserving surgery (BCS). A‐ii) Mammography images obtained using the intraoperative digital specimen mammography (IDSM) method. A‐iii) Nonpalpable breast lesions were wire‐localized. Adapted with permission.^[^ ^7c^ ^]^ Copyright 2007, Springer Nature. B) Ultrasound images of breast specimens with B‐i) transverse and B‐ii) longitudinal scans. The images show a hypoechoic mass close to the lateral margin, which was considered positive based on ultrasonographic assessment. Adapted with permission.^[^ ^8b^ ^]^ Copyright 2019, Elsevier.

SR is used for immediate assessment of tissue samples following biopsy or surgical excision. Conventional SR involves X‐ray imaging of excised tissue using mammography or a specimen radiography system (**Figure** **2**A). SR is performed on a nonpalpable lesion to exploit the X‐ray projection of the imaged tissue and produce contrast based on beam attenuation through the tissue. The standard of care involves using X‐ray projections to localize the center of the visible tumor and verify that the specimen contains the observed lesion. Three methods commonly used for preoperative localization of nonpalpable tumors are radioactive seed localization, wire‐guided localization, and radio‐guided occult lesion localization.^[^ ^7d,e^ ^]^

Compared to conventional SR, which needs significant time for transporting the surgical specimen from the operating room to the diagnostic imaging room for specimen radiography, intraoperative digital specimen mammography (IDSM), which can occur in the operating room, enables immediate radiography of the specimen. However, one study that compared tumor localization and margin estimation determined using conventional SR and IDSM reported that IDSM did not reduce overall operative times significantly, but it leads to a significant reduction in the positive margin rate.^[^ ^7c,f^ ^]^ However, clear margin width for specimen radiography has not yet been defined, and low sensitivity and specificity associated with conventional SR methods remain problematic.

### 3.2 Intraoperative Ultrasonography (IOUSG)

IOUSG imaging of specimen margins allows for visualization of structural features and associated heterogeneity (Figure 2B). Surgeons locate the tumor in the breast using ultrasound and compare findings with preoperative digital images. After excision, the surgeon can use ultrasound to examine the specimen ex vivo to confirm that it resembles the candidate lesion targeted preoperatively. In a multicenter, randomized controlled trial, IOUSG‐guided surgery significantly lowered the proportion of tumor‐involved resection margins compared with palpation‐guided surgery, thus reducing the need for re‐excision, mastectomy, and boost radiotherapy.^[^ ^7^, ^8^ ^]^

Since mammography has difficulty imaging through dense breast tissue, IOUSG may be a better alternative than specimen radiography. Moreover, IOUSG is much faster and more cost‐efficient than more commonly used radiography techniques.^[^ ^8d^ ^]^ However, the necessity of larger margins, low sensitivity, and a requirement to be scanned make IOUSG unlikely to be a full solution to the margin status problem.

## 4 Newly Emerging Diagnosis Methods

Given the disadvantages of pathologic analysis, newly emerging technologies have been developed that are advantageous in terms of speed, cost, and reliability, in addition to diagnostic accuracy. We describe methods based on computed tomography, MRI, spectroscopy, and chemical approaches. We discuss the advantages and disadvantages of these techniques in improving future real‐time intraoperative margin assessment in BCS.

### 4.1 Optical Coherence Tomography

**Figure 3:**
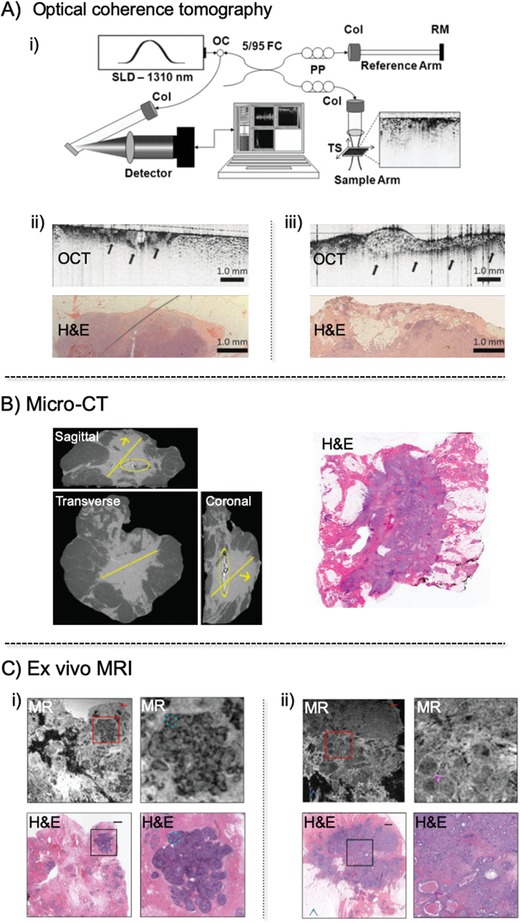
A‐i) Schematic of the spectral‐domain optical coherence tomography (OCT) system. Light from a superluminescent diode (SLD) is directed into an optical circulator (OC) and to a fiber coupler (FC), which splits 5% of the light to a reference arm mirror (RM) and 95% of the light to a sample arm containing focusing optics and an automated *x*–*y* translation stage (TS). Light is collimated through fiber collimators (Col). Reflected light from each arm is coupled through polarization paddles (PP), interfered within the fiber coupler, and spectrally dispersed onto a line camera. A‐ii,iii) OCT images of positive tumor margins show a distinct region with heterogeneous scattering. The corresponding histologic images with hematoxylin and eosin (H&E) staining confirmed the presence of positive margins. Adapted with permission.^[^ ^12a^ ^]^ Copyright 2009, American Association for Cancer Research. B) Micro‐computed tomography (Micro‐CT) images of a breast lumpectomy specimen with the largest dimension in the sagittal, transverse, and coronal planes. Arrow: a close margin (<0.1 cm) confirmed by histopathology; Circle: post‐biopsy clip. Adapted with permission.^[^ ^11b^ ^]^ Copyright 2001, British Institute of Radiology. C) Magnetic resonance images and corresponding light microscopy images with H&E stained of C‐i) DCIS, and C‐ii) IDC and DCIS. A magnified view (4× magnification) of the area within each red square is shown to its right. Left image scale bar = 1.25 mm. Adapted with permission.^[^ ^13b^ ^]^ Copyright 2011, Nature Publishing Group. DCIS = ductal carcinoma in situ; IDC = invasive ductal carcinoma.

Optical coherence tomography (OCT) is the optical version of ultrasound imaging, which applies a light wave instead of a sound wave in an entire live BCS specimen. The application of near‐infrared light leads to a high‐resolution, real‐time, multidimensional image of a cancer tissue sample up to 2 mm beneath the tissue surface (**Figure** **3**A).^[^ ^12a^ ^]^ The OCT light penetrates the entire live specimen, which is scattered back to the detector. Since cancer typically has a higher nuclear‐to‐cytoplasm ratio, higher cellular density, and higher nuclear density than fibrous and fatty tissue of normal mammary regions, cancer tissue has higher scattering properties. Adipocytes are imaged with depths of up to 2 mm, but tumors are imaged to depths of 200 to 1000 µm. Thus, normal gland tissue and cancer tissue are differentiated with OCT imaging.

Boppart and co‐workers have tested the surgical margins of lumpectomy specimens using their handmade, needle‐based OCT probe so that the depth of the field of the lens (1.47 mm) closely matches the penetration depth of OCT in the entire BCS specimen.^[^ ^12a^ ^]^ Their apparatus also includes a high‐resolution scanner providing enhanced images. When OCT‐based breast cancer surgical margin data were compared with data based on pathologic method, the sensitivity was 100% and specificity was 82% (9 true positives, 9 true negatives, 2 false positives, and 0 false negatives). These results show the potential of OCT imaging, but further examples and applications of OCT have not been reported. In addition, surgeons need training to be able to distinguish nonsuspicious from suspicious areas for margin management using OCT images with final histology as the reference standard.^[^ ^12b,c^ ^]^ Imaging protocols and evaluation criteria need to be standardized for real‐time intraoperative margin assessment in BCS.

### 4.2 Microcomputed Tomography (Micro‐CT)

Micro‐CT is a promising method for measuring the size of tumors in three dimensions in entire live BCS specimens (Figure 3B). Smith and co‐workers used a tabletop micro‐CT device, Skyscan 1173 (Skyscan, Kontich, Belgium), to measure the size of tumors in 50 invasive breast cancer specimens from 50 patients (42 IDC, 6 invasive lobular carcinoma (ILC), and 2 other invasive cancer).^[^ ^11b^ ^]^ To measure accuracy, they compared the micro‐CT data with data from preoperative mammography, ultrasound, MRI, and pathologic analysis (H&E staining). Compared with the largest dimension of the tumor on pathologic analysis, micro‐CT had the best correlation coefficient (*r* = 0.82, *p* < 0.001), followed by MRI (*r* = 0.78, *p* < 0.001), ultrasound (*r* = 0.61, *p* < 0.001), and mammography (*r* = 0.40, *p* < 0.01). In other words, mammography and ultrasound underestimate the largest tumor dimension, while MRI and micro‐CT overestimated it more frequently. Moreover, micro‐CT could provide 3D shape analysis with sufficient spatial conditions. Thus, it has the potential to be used as a predictor of which margins are most likely to be positive.^[^ ^11a^ ^]^

Future studies could make micro‐CT technology applicable for brief intraoperative margin assessment, although such macrosize analysis cannot diagnose the detailed morphological features of various cancers, which is the most critical factor for which new technologies should be developed for BCS.

### 4.3 Ex Vivo Magnetic Resonance Imaging (Ex Vivo MRI)

MRI has the potential to reveal characteristic pathologic features of both benign and malignant breast and lymphatic tissue (Figure 3C).^[^ ^13b^ ^]^ Agresti and co‐workers have reported that ex vivo MRI of entire live BCS specimens is a promising method for intraoperative diagnosis.^[^ ^13a^ ^]^ They injected gadolinium‐diethylenetriaminepentaacetic acid (Gd‐DTPA) as the MRI contrast reagent in 39 patients with breast cancer at 1 min before skin incision. After BCS was conducted with the support of preoperative MRI, the surgical specimens were further analyzed using ex vivo MRI with a dedicated surface coil and spectral attenuated inversion recovery sequences for suppression of fat signal intensity. All MRI enhancing lesions were included within the surgical specimen and efficiently visualized with ex vivo MRI. A significant advantage of ex vivo MRI is that the signals in the surgical sample were enhanced when compared with preoperative MRI signals. Ex vivo MRI visualized tumor regions more clearly than NBG and benign lesions. It is noteworthy that the 12 malignant tumors, including tumors from breast cancer type 1 susceptibility protein (BRCA1) mutation carriers, were all detected by ex vivo MRI but undetected with conventional preoperative imaging.

It should be noted again that MRI is an imaging modality for detecting macrosized cancers. The long scan times for MRI and high cost of the equipment might limit use of this method in settings such as small hospitals or developing countries. Therefore, an established procedure for re‐enhancing breast lesions within a surgical specimen would be a powerful technique for intraoperative diagnosis during BCS.

### 4.4 Radiofrequency Spectroscopy: MarginProbe Device

**Figure 4:**
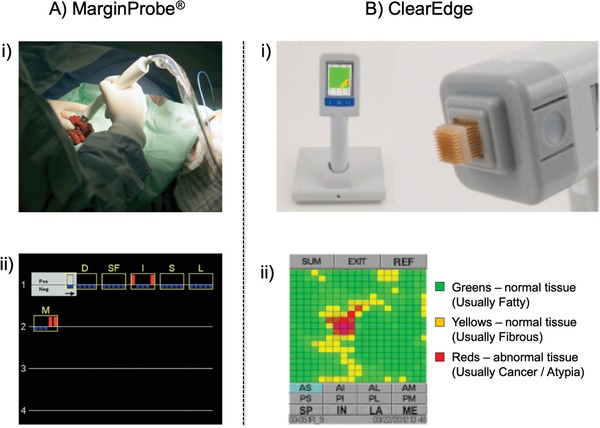
A‐i) Intraoperative use of the MarginProbe device with a breast lumpectomy specimen. Measurement is performed by applying the tip of the probe to a point on the resected lumpectomy specimen. During each measurement, radiofrequency signals are transmitted from the probe to the tissue, reflected, and collected by the console. The reflected signals are analyzed based on an algorithm. The device readings are displayed as “positive” or “negative.” A‐ii) The MarginProbe device output display for a typical patient. Data accumulate on the screen from left to right and from top to bottom. The most recent measurement is highlighted on the top left. Blue bars represent negative readings and red bars represent positive readings. Yellow frames and labels outline the margins from which readings are obtained. Adapted with permission.^[^ ^9a^ ^]^ Copyright 2008, Elsevier. B‐i) The ClearEdge device is a portable, battery operated, handheld imaging device equipped with a sterile head for use in a single patient. B‐ii) ClearEdge color‐coded image display. Each scan produces a color‐coded image on the device's screen display. Green and yellow pixels are considered to be characteristic of normal tissue and red pixels are considered to indicate abnormal tissue. Adapted with permission.^[^ ^10^ ^]^ Copyright 2016, Elsevier.

MarginProbe (Dune Medical Devices Ltd, Caesarea, Israel) was developed to measure the local electrical properties of lumpectomy margins in the radiofrequency range (**Figure** **4**A). Such electrical properties depend on the membrane potential, nuclear morphology, cellular connectivity, and vascularity of live tissues. Thus, cancerous and normal regions could be efficiently discriminated. The threshold between positive and negative margins has been already determined by comparing data to pathologic results. Therefore, if applicable, surgeons could use this method during routine operations. Practically, six surfaces of the main lumpectomy specimens can be measured with MarginProbe within 20 min (five to eight times for each surface). Many clinical trials have been completed with the MarginProbe device, including the MAST study in Israel, the US Pivotal Study in the United States, and a multicenter study in Germany.^[^ ^9a,b^ ^]^

Schnabel and co‐workers conducted prospective clinical trials on real‐time intraoperative assessment of lumpectomy margins in 596 patients with breast cancer.^[^ ^9c^ ^]^ After removing the margins during surgery, patients were randomized to the device or control arms. In the device arm, MarginProbe was used to examine the main lumpectomy specimen and guide additional direct excision of positive margins. In the control arm, the cancer regions to be removed were evaluated by surgeons without any devices, the current standard of care in most hospitals in America and Europe. The false‐negative rates were 24.8% and 66.1% and the false‐positive rates were 53.6% and 16.6% in the device and control arms, respectively. Based on this intraoperative analysis, 62% of the main positive specimens were in the device arm compared with 22% in the control arm (*p* < 0.001). As a result, 19.8% of patients in the device arm underwent a re‐excision procedure compared with 25.8% in the control arm. As the authors note, the adjunctive use of the MarginProbe device during BCS would help with intraoperative cancer assessment, thus, reducing the need for re‐excision. However, while performing BCS, the surgeon should also consider adverse cosmetic effects.

These studies have led to the Food and Drug Administration (FDA) approval of MarginProbe as a device to help surgeons identify positive margins. It is approved for use in the United States to assess the adequacy of surgical breast margins intraoperatively. However, the MarginProbe device, which works based on user‐guided spot scanning, also has severe drawbacks such as low sensitivity, low specificity, and high false‐positive rates.

All these new technologies described to date (OCT, micro‐CT, ex vivo MRI, and MarginProbe) can measure the macro‐size of tumor tissues, but cannot analyze cancer morphology or provide accurate localization of the lesion in live tissues. Regarding practical use during intraoperative BCS, it is necessary to detect cancer at the micro‐size level in lumpectomy margins, which can be accomplished with the standardized pathologic methods. The following sections discuss trials addressing these challenges.

### 4.5 Bioimpedance Spectroscopy: ClearEdge Device

ClearEdge is a handheld portable imaging device that uses bioimpedance spectroscopy to detect differences in tissue dielectric properties of the resected specimen during BCS (Figure 4B). This device performs a baseline measurement from the patient's NBG and uses normalized data to scan all margins of the excised tissue specimen. A randomized trial of intraoperative margin status assessment during BCS reported that the re‐excision rate was lower for patients treated with ClearEdge versus specimen radiography. In addition, ClearEdge can complete a full scan in less than 5 min. Bioimpedance spectroscopy is promising and straightforward. However, the lack of sensitivity and specificity still needs to be addressed.^[^ ^10^ ^]^

### 4.6 Raman Spectroscopy Combined with Autofluorescence Microscopy

Raman spectroscopy measures the vibrational frequencies of molecules in tissues that could be excited by a laser. Although Raman spectroscopy has been used to diagnose breast cancer with high sensitivity and specificity, one drawback of this method is the amount of time required. Raman spectroscopy cannot image small areas of residual cancer in full tissue sample surface areas of BCS specimens with sufficient accuracy within the limited timeframe possible for intraoperative diagnosis.

**Figure 5:**
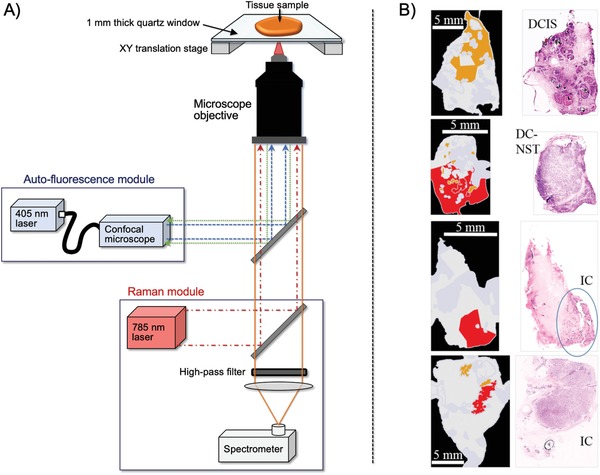
A) Multimodal spectral histopathology (MSH) instrument consist of an inverted optical microscope with an integrated Raman spectrometer (excitation 785 nm, detection Raman shift range, 600–1800 cm^−1^) and a confocal autofluorescence module (excitation 405 nm, detection range 450–520 nm). B) Images of breast cancer tissue detected with MSH. The corresponding histologic images with hematoxylin and eosin (H&E) staining confirmed the presence of positive margins. Adapted with permission.^[^ ^17b^ ^]^ Copyright 2018, Springer Nature. DCIS, ductal carcinoma in situ; DC‐NST, ductal carcinoma of no special type; IC, invasive carcinoma.

Notingher and co‐workers recently developed a multimodal imaging technique combining tissue autofluorescence (excitation at 405 nm, detection at 450–520 nm) and Raman spectroscopy (excitation at 785 nm, Raman shift detection at 600–1800 cm^−1^), which they called multimodal spectral histopathology (**Figure** **5**).^[^ ^17b^ ^]^ They extensively optimized the sampling and data processing algorithms to use autofluorescence images to guide Raman measurements and achieve high spatial and spectral information in just 12–24 min, even when analyzing large tissue surfaces up to 4 cm ×  6.5 cm. Analysis of 121 surgical marigin specimens from 107 patients, although not under real‐time intraoperative assessment conditions, could discriminate IDC and DCIS from NBG with 95% sensitivity and 82% specificity. They reported that cancer lesions smaller than 1 mm^2^ could be analyzed. However, these analyses used a low‐power field, and detailed morphological analysis with a high‐power field, i.e., micro‐sized analysis, has not been reported.

Mahadevan‐Jansen and co‐workers investigated the feasibility of a 3D scanner that relies on Raman spectroscopy to assess all of the margins of a resected specimen within a clinically feasible time. They demonstrated the potential of this device for automated breast tumor margin assessment, which could minimize repeat invasive surgeries.^[^ ^17a^ ^]^

### 4.7 Ultraviolet‐Photoacoustic Microscopy (UV‐PAM) and Microscopy with Ultraviolet Surface Excitation (MUSE)

Recently, an innovative microscopic technique to image cellular structures and their organization in tissue samples, i.e., at the morphological and cellular levels, has been developed. Photoacoustic tomography is a rapidly growing imaging modality that can provide volumetric images with high resolution. Photoacoustic tomography, which is based on optical absorption contrast with appropriate wavelength illumination, is highly specific for a particular target within cells. Cheng and co‐workers reported a multispectral photoacoustic tomography system for breast cancer margin management using fat and hemoglobin as contrasts. The system can analyze tissue depths of ≈3 mm with an axial resolution of ≈125 µm.^[^ ^14a^ ^]^

**Figure 6:**
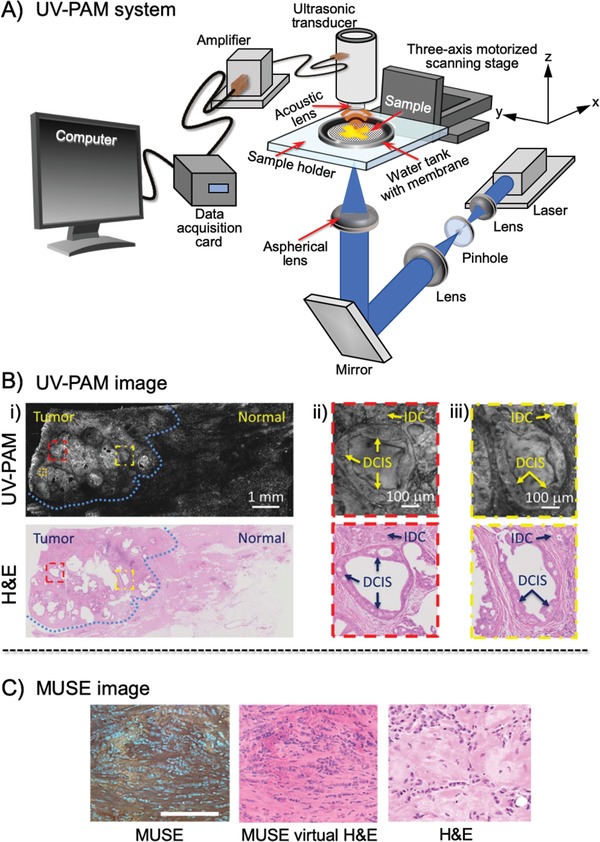
A) Schematic of the ultraviolet‐photoacoustic microscopy (UV‐PAM) system for surgical margin imaging. A pair of lenses and a pinhole filter and expand the UV laser beam. The aspherical lens focuses the beam onto the bottom of the breast tissue sample placed inside a water tank on top of a sample holder. Some acoustic waves propagate through the tissue and reach a focused ultrasonic transducer, which is then transduced into an electric signal, amplified, and recorded by a data acquisition card. An image from the measured data is shown on a computer screen. B‐i) UV‐PAM image of a fixed, unprocessed breast tumor and its corresponding histologic image with hematoxylin and eosin (H&E) staining acquired after processing, sectioning, and staining the excised breast tissue. The blue dashed lines indicate the interface between normal and tumor regions. Scale bar =  1 mm. B‐ii) Magnified UV‐PAM images and images with H&E staining of the red dashed regions in B‐i. Scale bar =  100 µm. B‐iii) Magnified images and images with H&E staining of the yellow dashed regions in B‐i. Scale bar =  100 µm. Adapted with permission.^[^ ^14b^ ^]^ Copyright 2017, American Association for the Advancement of Science. C) Left: Microscopy with ultraviolet surface excitation (MUSE) fluorescence images from the cut surfaces of formalin‐fixed invasive lobular carcinoma (ILC) tissues briefly stained with Hoechst stain, rhodamine, eosin, and propidium iodide, captured with a color camera after white balancing. Middle: Images converted to virtual H&E staining. Right: digital images captured using a whole‐slide scanner from conventional slides with H&E staining of the same specimens after paraffin embedding and sectioning. Scale bar =  100 µm. Adapted with permission.^[15]^ Copyright 2017, Springer Nature.

Wang and co‐workers developed PAM, which was combined with UV laser illumination.^[^ ^14b^ ^]^ The advantage of UV laser illumination is that it could highlight the nuclei so that their PAM technique could provide images similar to those obtained with H&E staining, a conventional and reliable method used for real‐time intraoperative margin assessment in BCS (see Section 2.1). In their housemade photoacoustic microscopy device depicted in **Figure** **6**A, a UV laser beam was designed to focus onto breast tissue specimens. Laser‐induced rapid thermoelastic expansion induces acoustic waves that could then be transduced into electric signals, amplified, and recorded. With optimized experimental parameters and automated algorithms, this method could accurately compute and diagnose the size, internuclear distance, and packing density of nuclei. It provides images similar to those obtained with pathologic techniques (Figure 6B). However, the analysis requires a few hours; further optimization is necessary before use in real‐time intraoperative margin assessment in BCS.

Levenson and co‐workers reported that MUSE could also generate shape and color‐contrast information (Figure 6C).^[^ ^15^ ^]^ MUSE exploits the low‐penetration depth of UV light to excite fluorophores at the surface of stained tissue. MUSE relies on UV wavelengths of ≈280 nm to restrict the excitation of conventional fluorescent stains to tissue surfaces. MUSE has the potential to improve the efficiency of patient care in both state‐of‐the‐art and low‐resource settings and to provide opportunities for rapid histology.

### 4.8 Chemical Probes

Chemists are currently actively investigating chemical probes to diagnose cancer based on fluorescence properties that are highly sensitive and use the optimal wavelength to avoid background signals from live samples based on rational design. In general, these strategies use the fluorescence switching properties of chemical probe structures, which are activated by cancer‐specific enzymes. In addition, an alternative approach of applying synthetic transformation selectively in cancer cells has also been reported recently.

#### 4.8.1 Fluorescence “Switch‐On” Probe by Cancerous Enzymatic Activity

**Figure 7:**
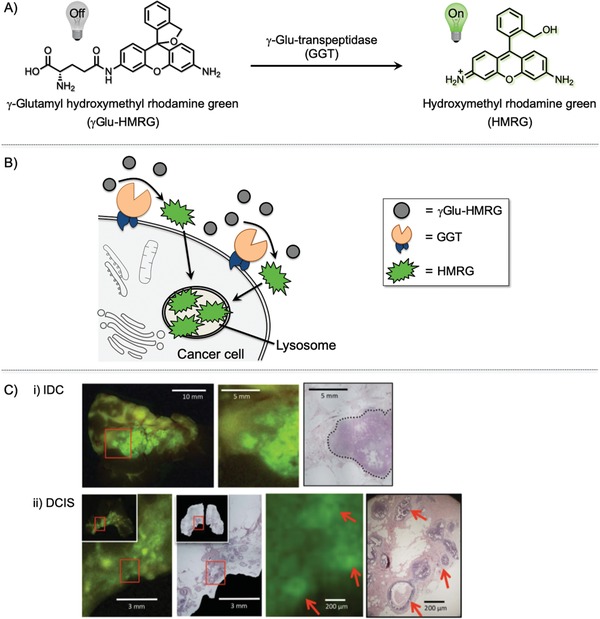
A) Explicit activiation of the γ‐glutamyl hydroxymethyl rhodamine green (γGlu‐HMRG) probe by γ‐glutamyltranspeptidase (GGT). B) Mechanism of γGlu‐HMRG activation. γGlu‐HMRG reacts with upregulated GGT anchored on the plasma membrane of cancer cells. This interaction enzymatically cleaves the probe to yield a highly fluorescent and membrane‐permeable product, hydroxymethyl rhodamine green (HRMG), which accumulates in the lysosomes of cancer cells. HMRG does not covalently attach to the inside of the lysosome; thus, it can be excreted out of the cell again and affect the surrounding cells. C) Comparison of fluorescence localization with pathologic hematoxylin and eosin (H&E) staining. C‐i) The cancer region is enclosed by a dotted line in the image with H&E staining. C‐ii) Red arrows show fluorescence‐positive areas and malignant lesions. Adapted with permission.^[^ ^16a^ ^]^ Copyright 2011, Nature Publishing Group.

Urano and co‐workers are pioneers in the development of fluorescence “switch‐on” probes. They have designed various innovative fluorogenic probes that could be activated by a cancer‐specific enzyme.^[^ ^16a^ ^]^ For example, γ‐glutamyltranspeptidase (GGT) is overexpressed on cancer cell surfaces but not on normal cell surfaces. They developed γ‐glutamyl hydroxymethyl rhodamine green (γGlu‐HMRG), which is fluorescently inactive but can be selectively activated by an enzymatic reaction on cancer cell surfaces (**Figure** **7**). γGlu‐HMRG has been used to discriminate IDC and DCIS from NBG. The sensitivity and specificity of this method was 92% and 94%, respectively. This method could detect cancer regions that are smaller than 1 mm. The fluorescence signals were obtained within 5 min after treatment of live tissues.

However, their method relies on a time‐dependent increase in fluorescence. Thus, sample‐to‐sample reproducibility, i.e., cancer selectivity due to high fluorescence background and fluorescent spreading, needs to be improved before use during actual BCS. More importantly, the fluorescently activated molecule is generated on the cell surface, not in the cancer cells. Thus, the fluorescence gradually spreads during the analysis and fluorescence images become ambiguous. They compared their data with those of pathologic analysis, but morphological information has not been deduced from their fluorescence images. These methods are thought to be useful for actual BCS; however, since the method cannot be used to determine cancer morphology in live tissues, it is unlikely to replace pathologic methods used on frozen tissue specimens.

#### 4.8.2 Fluorescence “Switch‐On” Probe Activated upon Cancerous Protein Binding

**Figure 8:**
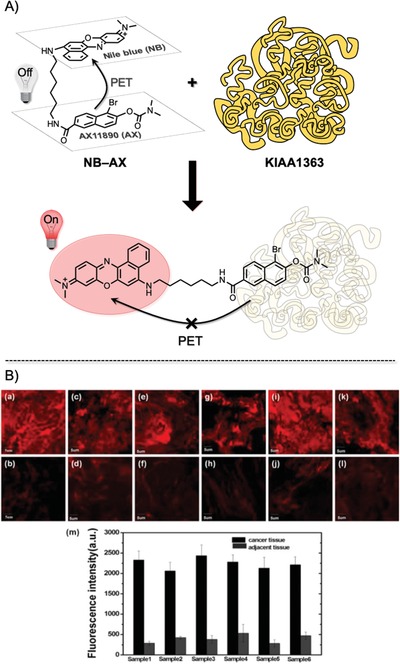
A) The NB‐AX probe is composed of Nile blue (NB) dye and AX11890, a selective inhibitor of enzyme KIAA1363, linked by a flexible linear hexanediamine chain. Free NB‐AX (top) is in a folded structure and has weak fluorescence quenched by the photoinduced electron transfer (PET) effect. However, in the presence of KIAA1363, the AX11890 portion of the probe selectively binds to the enzyme, and NB‐AX becomes unfolded (bottom). Consequently, the PET effect stops, and the fluorescence signal of NB‐AX increases immediately. B) Fluorescence images of NB‐AX in human breast cancer tissue (top) and adjacent tissue (bottom). Live tissues were stained with 10 × 10^−6^ m of NB‐AX for 5 min. a,b), c,d), e,f), g,h), i,j), and k,l) belong to experiments labeled samples 1–6, respectively. Images were generated using an excitation wavelength of 635 nm and were captured at 655–755 nm. Quantitative image analysis of average fluorescence from seven areas in each sample image (m). Adapted with permission.^[^ ^16b^ ^]^ Copyright 1996, Royal Society of Chemistry.

Fan et al. developed a near‐infrared (NIR) fluorescent probe that can be efficiently activated by interaction with neutral cholesteryl ester hydrolase 1 (KIAA1363), which is highly overexpressed in various invasive breast cancers (**Figure** **8**).^[^ ^16b^ ^]^ The AX11890 compound, which is the ligand of KIAA1363, is linked with Nile blue (NB) to quench the fluorescence of AX11890 with an efficient photoinduced electron transfer (PET) mechanism in aqueous buffer solution. However, when the “silent” probe interacts with KIAA1363, AX11890 becomes separated from the NB dye by a certain distance and recovers NIR fluorescence (“switch‐on”). This probe was found to be highly selective for KIAA1363 among the biomolecules investigated. The fluorescence recovery was quick, with a detection limit of 0.58 µg mL^−1^ (3δ/*k*). The probe was used to selectively stain human breast cancer tissue within 5 min. Red fluorescent signals could be obtained at a depth of 0–980 µm with excitiation at 635 nm (emission recorded at ≈700 nm). This probe has excellent NIR fluorophore properties for assessing tumors inside thick tissue samples as well as reducing background signals in human tissue samples. However, the ambiguity and generalized expression of the KIAA1363 protein in heterogeneous cancer tissues from human patients does not motivate surgeons to use this method during BCS.

It should be noted that, during BCS, various cancerous tissues from patients with breast cancer need to be stained or imaged, not those from cell lines or animal models in experimental laboratories that always express specific tumor‐associated enzymes or antigens (in the case of antibodies). Previous trials have failed in immunostaining clinical tumor samples for this reason. Currently, none of the newly emerging fluorescent probes, which rely on cancer‐specific proteins, antigens, or enzymes, have been successfully applied to real‐time intraoperative margin management in BCS.

#### 4.8.3 New Modality Involving Fluorescent Labeling of Live Cancerous Tissues through an “In‐Cell” Cascade Reaction with Acrolein

As discussed above, the main problem associated with fluorescence‐based methods is generalizability to human patients, because the target enzymes or proteins that could activate the fluorescent switch might not be expressed in all type of cancers. In addition, the activated fluorescence on cancer tissues can only be evaluated in a time‐dependent manner, which leads to issues with reproducibility. Therefore, unlike conventional H&E sectioning analysis, morphological analysis of cancer, which is the critical analysis in BCS, cannot be performed, although the fluorescent techniques are up‐and‐coming in terms of sensitivity and specificity. On the other hand, a novel fluorescence‐based concept using the acrolein‐initiated cascade reactions in live breast cancer tissues has been recently developed.^[^ ^16^, ^22^ ^]^

**Figure 9:**
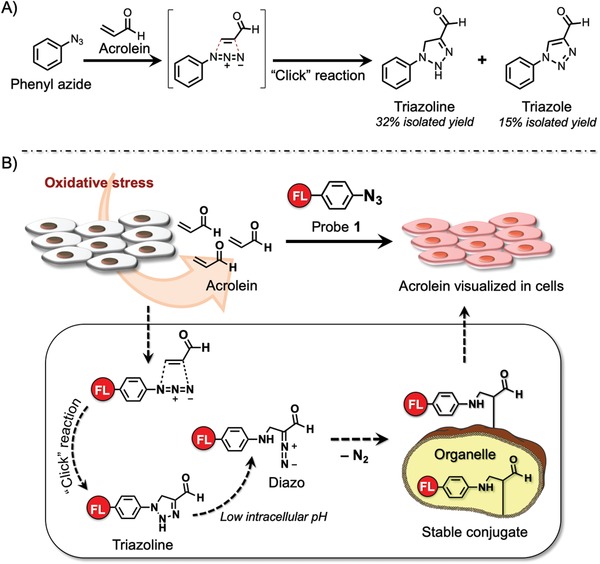
A) Phenyl azide reaction with acrolein. B) “Click‐to‐sense” method and mechanism. Fluorescence‐labeled phenyl azide smoothly reacts with acrolein generated by cancer cells through a 1,3‐dipolar cycloaddition reaction (azide/acrolein click reaction). The triazole decomposes into diazo compounds, which react with cellular constituents to anchor the fluorescence label within cells. The concentration of acrolein is analyzed based on the fluorescence readout at the whole‐cell level. FL = tetramethylrhodamine (TAMRA).

Acrolein, a highly toxic α,β‐unsaturated aldehyde,^[^ ^23^ ^]^ has been reported to be a biomarker associated with various type of disorders related to oxidative stresses. Acrolein is produced through the enzymatic oxidation of polyamines^[^ ^24^ ^]^ and during reactive oxygen species (ROS)‐mediated oxidation of highly unsaturated lipids.^[^ ^25^ ^]^ The authors developed an “in‐cell” reactivity probe **1** for detecting acrolein based on an acrolein/azide click reaction (**Figure** **9**A).^[^ ^26^ ^]^ In contrast to previously reported methods for detecting acrolein,^[^ ^27^ ^]^ this method can sensitively detect the presence of acrolein within live cells even at the nM level. The azide functionality in probe **1** participates smoothly in the 1,3‐dipolar cycloaddition reaction with acrolein, generated by cells, to give triazoline derivatives (Figure 9B). Under conditions of lower intracellular pH, the triazoline derivatives then decompose into corresponding diazo compounds, which are immediately and nondiscriminately conjugated with cell constituents to anchor fluorescence within the cell. Therefore, in clear contrast to previously developed fluorescence switch‐on methods, cancer could be labeled and imaged at the cellular level. This new method is noteworthy for its simplicity.

**Figure 10:**
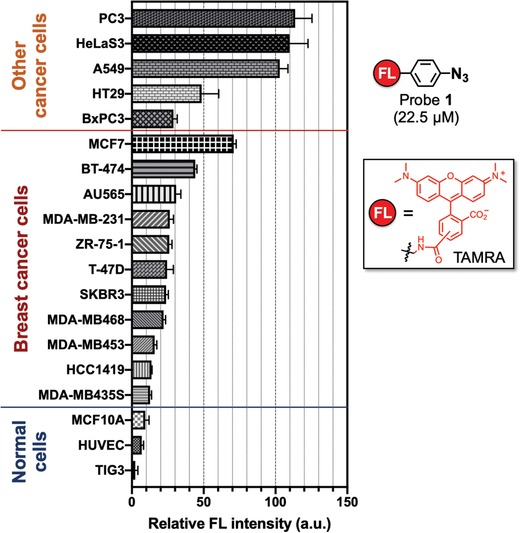
Intensity of fluorescence observed for cell lines over 22.5 × 10^−6^ m of probe **1**. Three normal cell lines were tested, from bottom to top: TIG3 (normal human diploid cells), HUVEC (human umbilical vein endothelial cells), and MCF10A (normal human mammary cells). Eleven breast cancer cell lines were tested, from bottom to top: MDA‐MB435S (ER^−^/PR^−^/HER2^−^), HCC1419 (ER^−^/PR^−^/HER2^+^), MDA‐MB453 (ER^−^/PR^−^/HER2^+^), MDA‐MB468 (ER^−^/PR^−^/HER2^−^), SKBR3 (ER^−^/PR^−^/HER2^+^), T‐47D (ER^+^/PR^+^/HER2^−^), ZR‐75‐1 (ER^+^/PR^+^/^−^/HER2^−^), MDA‐MB‐231 (ER^−^/PR^−^/HER2^−^), AU565 (ER^−^/PR^−^/HER2^+^), BT‐474 (ER^+^/PR^+^/HER2^+^), and MCF7 (ER^+^/PR^+^/HER2^−^). Five other cancer cell lines, from bottom to top: BxPC3 (human pancreatic cancer cells), HT29 (human colon cancer cells), A549 (adenocarcinomic human alveolar basal epithelial cells), HeLa S3 (human cervical cancer cells), and PC3 (human prostate cancer cells). The intensity of fluorescence was normalized to the intensity detected from 10000 cells. The immunoprofile of the breast cancer subtypes is shown in parentheses. ER, estrogen receptor; PR, progesterone receptor; HER, human epidermal growth factor receptor 2; FL, tetramethylrhodamine (TAMRA).

It has been reported that cancer cells are under oxidative stress conditions associated with increased production of ROS.^[^ ^28^ ^]^ Based on previous report that cancer cells produce acrolein,^[^ ^27^ ^]^ we have used probe **1** to label different cancer cells (**Figure** **10**).^[^ ^16^, ^22^ ^]^ In other words, cancer cells produce a significant amount of acrolein, and this cellular acrolein could be used as a new cancer marker.

**Figure 11:**
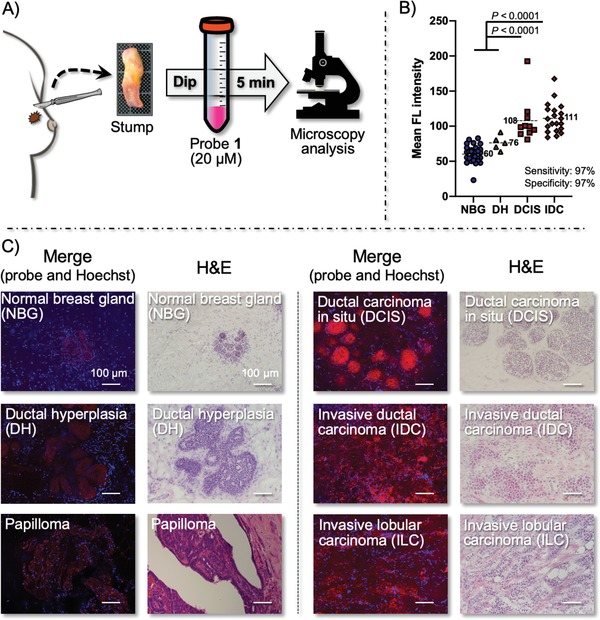
A) Schematic of the procedure for labeling of breast cancer stumps with probe **1**. B) Morphology of normal breast gland (NBG), ductal hyperplasia (DH), papilloma, ductal carcinoma in situ (DCIS), invasive ductal carcinoma (IDC), and invasive lobular carcinoma (ILC) labeled with 20 × 10^−6^ m of probe **1** at 200× magnification. The cancer morphology detected was consistent with the morphology in images with hematoxylin and eosin (H&E) staining in frozen sections prepared from the same anonymized tissue samples. Adapted with permission.^[^ ^16c^ ^]^ Copyright 2014, John Wiley and Sons Inc.

We then applied the probe **1** to live tissue samples (20 IDC,10 DCIS, 30 NBG, and 5 DH) from patients with breast cancer who underwent breast surgery at Osaka University Hospital, in Osaka, Japan from March 2017 to March 2018. The live tissues were cut into a flat surface, immersed into a 20 × 10^−6^ m solution of probe **1** for 5 min, and rinsed with buffer (**Figure** **11**A).^[^ ^16c^ ^]^ The resulting tissues were then directly analyzed using a Keyence BZ‐X710 fluorescence microscope equipped with an optical sectioning system to obtain both gross images and double fluorescence‐stained images.^[^ ^29^ ^]^ The mean fluorescence intensity of IDC and DCIS samples were statistically significantly higher than that of DH and NBG samples (Figure 11B), with sensitivity and specificity of 97% and 97% for tumors, respectively. Since all cancerous cells produce a high level of acrolein, probe **1** could label breast cancer tissues with a similar mean fluorescence intensity, regardless of their subtypes such as estrogen receptor (ER) or progesterone receptor (PR) or human epidermal growth factor receptor 2 (HER2) status.

Of note, when the fluorescence‐labeled IDC and DCIS images were magnified by 200×, the morphology of cancers could be imaged and discriminated (Figure 11C). This method could visualize the morphology of IDC and DCIS as well as ILC, DH, and papilloma. Blind testing of the fluorescent images by pathologists with an anonymized data set are in good agreement with H&E stained images of the same sections. This notable feature of probe **1**, which enables the visualization of cancer morphology, comes from its ability to selectively label the cellular contents only in cancer cells within live tissues (Figure 9B). Thus, this click‐to‐sense method, which is not a conventional pathologic method, can accurately identify residual carcinoma by identifying morphology, even at the cellular level. Moreover, this chemistry‐based diagnosis method only requires 5 min. With this promising method, live tissue morphology in patients with breast cancer can be easily identified, providing future support for BCS.

## 5 Meta‐Analysis: What Method Is Better?

A systematic Review of 106 scientific papers from 1995 to July 2016 concluded that frozen section analysis, imprint cytology, and ultrasound‐guided lumpectomy can lower positive margin rates compared with palpation guidance.^[^ ^6c^ ^]^ However, the effects of specimen radiography on positive margin rates have not been evaluated to date. Cavity shave margins and the MarginProbe device similarly decrease the positive margin rate, but at the same time, we should take into account that these methods might be associated with negative cosmetic effects.^[^ ^6c^ ^]^

Alternatively, a meta‐analysis of 35 studies on intraoperative margin assessment in BCS from a search conducted in January 2016 focused on cancer sensitivity and specificity and AUC with frozen section analysis (*n* = 9), imprint cytology (*n* = 11), IOUSG (*n* = 4), SR (*n* = 9), and OCT (*n* = 3).^[^ ^6b^ ^]^ Frozen section analysis (sensitivity, 86%; specificity, 96%; AUC, 0.96) and imprint cytology (91%, 95%, 0.98) have excellent diagnostic accuracy compared with IOUSG (59%, 81%, 0.78), SR (53%, 84%, 0.73), and OCT (85%, 87%, 0.88). OCT seems to have high diagnostic accuracy, but data are scant (*n* = 3). A global standard for this method and analysis should be established for wider applicability in intraoperative margin assessment.^[^ ^6b^ ^]^

## 6 Summary and Future Prospects

As discussed in the introduction, intraoperative pathologic diagnostic techniques such as frozen section analysis and imprint cytology have gradually gain recognition as conventional tools in BCS that lower the positive margin rate and eliminate repeat surgery, as demonstrated by a number of studies and meta‐analyses. However, practically, only a small number of hospitals, mainly in developed countries, routinely use these methods. Unfortunately, these methods cannot be used worldwide with BCS. Tedious pathologic procedures, e.g., making frozen sections and collaborating with pathologists in close proximity to operating rooms, require significant time, ≈30 min for a single analysis. This workload does not motivate surgeons to perform additional intraoperative diagnostic procedures. These problems have been the bottleneck for a long time for BCS in almost all hospitals, regardless of how many patients with breast cancer are waiting for treatment. To circumvent such problems and provide patients with improved surgical options and outcomes and to avoid re‐excision, two points should be taken into consideration. One is the development of nonpathologic methods for intraoperative use with consideration of resolution, sensitivity, specificity, speed, convenience, and cost. Another is the development of new imaging algorithms to standardize data for analyzing positive margins, as represented by automatic deep learning and AI algorithms without the need for judgment by well‐trained pathologists.

Regarding the new imaging methods, in this Review we introduced various techniques and trials involving OCT,^[^ ^12^ ^]^ micro‐CT,^[^ ^11^ ^]^ ex vivo MRI,^[^ ^13^ ^]^ MarginProbe,^[^ ^9^ ^]^ ClearEdge,^[^ ^10^ ^]^ UV‐PAM,^[^ ^14^ ^]^ MUSE,^[^ ^15^ ^]^ a multimodal imaging technique combining tissue autofluorescence and Raman spectroscopy,^[^ ^17^ ^]^ and chemistry‐based fluorescent methods.^[^ ^16^ ^]^ It is essential to mention that analyzing human breast cancer tissues is more complicated compared with other cancers. For instance, gastrointestinal cancers, such as gastric cancer, colorectal cancer, and pancreatic cancer, mainly consisted of adenocarcinoma. By contract, breast cancer consists of multiple histopathologic features and heterogeneous borderline lesions. Analyzing and classifying breast cancer pathology is undoubtedly the most important and challenging than classifying other cancers. Methods that do not evaluate the morphology of breast cancer tissue during BCS, which corresponds to visualizing cancer cells in live tissues, could hardly be used for intraoperative diagnostic purposes. While the apparatus and techniques for visualizing the size of cancer lesions on the macro‐ level can be used for intraoperative diagnosis of peritoneal metastasis during laparotomy for gastrointestinal cancer, it is difficult to apply them to breast cancer surgery. Thus, despite being significant technological advances, these new techniques and time‐dependent switch‐on fluorescence probes need improvement.

For this prospective, our click‐to‐sense method can rapidly discriminate between cancer and normal cells, requiring only staining of live tissues for 5 min. Moreover, it can visualize cancer morphology and provide localization in a way almost equivalent to frozen sectioning.^[^ ^16c^ ^]^ Our method is not affected by sample conditions, fluorescence background, or fluorescence spreading. Moreover, it is not dependent on the timing of enzymatic reactions or enzyme expression by cancer cells since it focuses explicitly on the overexpression of endogenously generated acrolein in various cancer cells. The rapid in‐cell cascade reactions selectively anchor the fluorescence label onto the cellular constituents in tumors. Our chemistry‐based method has the potential to become a new highly selective margin management method for live tissues; it could be used as a discriminative, low‐cost, and easy‐to‐perform method for cancer sensing during surgery. The technique will be confirmed in a prospective clinical study including intraoperative assessment of resection stumps in patients with breast cancer. The clinical significance of our probe in evaluating morphological and pathologic features deserves further investigation in hospitals worldwide.

It would be useful to consider applying automatic deep learning and AI algorithms. As discussed in Section 2.3., AI has shown potential usefulness as a diagnostic tool in pathologic diagnosis; AI outperformed pathologists in diagnosing even small amounts of cancer in frozen sections that had spread to lymph nodes in patients with breast cancer. Once efficient intraoperative techniques have been developed, then AI can automatically and rapidly evaluate cancer margins. Of note, our click‐to‐sense method could be used to diagnose cancer tissues during BCS more quickly than the conventional H&E method. Combining this method with AI, namely, analyzing the fluorescent morphological features by established AI algorithms, would lead to the next generation of real‐time intraoperative assessment.

While intraoperative diagnosis of breast cancer, a heterogeneous disease, is one of the most challenging endeavors, close collaboration among scientists in different fields is worthwhile for improving our quality of life.

A.R.P. and T.T. contributed equally to this work. This work was supported by Grants‐in‐Aid for Scientific Research from the JSPS KAKENHI Grant Numbers JP18K14341, JP18K07265, and JP15H05843 in Middle Molecular Strategy.

## Conflict of Interest

The authors declare no conflict of interest.
